# Avian Influenza H5N1 and Healthcare Workers

**DOI:** 10.3201/eid1107.050070

**Published:** 2005-07

**Authors:** Constance Schultsz, Vo Cong Dong, Nguyen Van Vinh Chau, Nguyen Thi Hanh Le, Wilina Lim, Tran Tan Thanh, Christiane Dolecek, Menno D. de Jong, Tran Tinh Hien, Jeremy Farrar

**Affiliations:** * Oxford University Clinical Research Unit at the Hospital for Tropical Diseases, Ho Chi Minh City, Vietnam;; † Pediatric Hospital Number 2, Ho Chi Minh City, Vietnam;; ‡ The Hospital for Tropical Diseases, Ho Chi Minh City, Vietnam;; § Department of Health, Hong Kong Special Administrative Region, People's Republic of China

**Keywords:** Avian influenza A virus, health care workers, infection control

**To the Editor:** Since January 2004, 35 human cases of avian influenza A virus H5N1 have been reported in Vietnam. Human-to-human transmission of H5N1 is a major concern, particularly because of reported family clustering (1). Two probable cases of human-to-human transmission were recently reported from Thailand (2), and evidence for human-to-human transmission was found in the 1997 Hong Kong outbreak (3). We evaluated healthcare workers exposed to 2 patients (patients 5 and 6 [1], referred to as patients A and B, respectively, in this article) with H5N1 infection, confirmed by polymerase chain reaction (PCR), to determine the potential risk for nosocomial human-to-human transmission of H5N1.

Patient A was admitted to a general ward of a pediatric hospital in Ho Chi Minh City on January 15, 2004, on day 8 of illness; no infection control measures were taken at that time. On January 18, 2004, she was transferred to the intensive care unit (ICU). Eight hours after ICU admission, limited infection control measures were implemented: the patient was transferred to a single room, and healthcare workers were required to use disposable surgical masks and gloves and wear nondisposable gowns. However, because resources were limited, each healthcare worker wore only 1 glove. On January 23, patient A was transferred to another hospital.

Patient B was admitted to the infectious diseases ward of the pediatric hospital on January 19, 2004, on day 6 of illness; he was transferred to the ICU after 4 hours and stayed there until he died on January 23. Infection control measures were implemented 2 days after ICU admission; these measures were similar to those taken for patient A except that no single room was available.

From January 25 to 27, 2004, a nasal swab specimen and baseline serum sample were collected from healthcare workers at the hospital; each worker also completed a questionnaire. On February 9 and 10, follow-up serum samples were collected. Nasal swab samples were tested by reverse transcription (RT)-PCR to detect the H5 gene (1). Paired serum samples were subjected to enzyme-linked immunosorbent assay (ELISA) (Virion/Serion, Würzburg, Germany) to detect immunoglobulin G against the nucleoprotein of influenza A; samples were also subjected to an H5-specific microneutralization assay (4).

Of 62 healthcare workers involved in caring for patient A, patient B, or both, 60 (97%) provided both samples and questionnaires: 16 who cared for patient A on the general ward, 33 who cared for patients A and B in the ICU, and 11 who cared for patient B on the infectious diseases ward or who were consulted for diagnostic or clinical procedures involving either patient. Characteristics of the workers and their exposures are shown in the Table.

**Table:** Characteristics of 60 healthcare workers exposed to avian influenza patient A, patient B, or both

Characteristic*	No. (%)
Median age (y) (n = 60)	33 (range 22–54)
Male/female (n = 60)	14/46
Occupation (n = 60)	
Nurse	28 (46.7)
Physician	10 (16.7)
Cleaner	9 (15.0)
Technician (laboratory/radiology)	9 (15.0)
Other	4 (6.7)
Flulike illness in preceding 2 weeks (n = 49)	6 (12.0)
Contact with poultry or birds (healthy or sick) (n = 59)	2 (3.4)
Recent travel to Mekong Delta (n = 59)	5 (8.4)
Duration of exposure (n = 59)	
<12 h	30 (50.8)
12–36 h	18 (30.5)
>36 h	11 (18.6)
Contact with secretions (n = 59)	
Yes	15 (25.4)
No	15 (25.4)
Don't know	29 (49.2)

*n indicates number of healthcare workers for which data were available.

The median time between last exposure and collection of the nasal swab and the baseline serum samples was 7 days (range 2–12 days). The median time between last exposure and collection of the follow-up serum sample was 21 days (range 17–26 days). All 60 nasal swab samples were negative by RT-PCR. Paired serum samples were available from 46 healthcare workers, and 42 were negative in the influenza A–specific ELISA, 2 reacted with a negative-to-borderline response, 1 had a borderline-to-positive response, and 1 had 2 positive responses. A positive response indicates recent infection. All paired serum samples, 12 additional baseline samples, and 2 additional follow-up samples were negative in the H5-specific microneutralization assay. None of the paired samples from 4 healthcare workers that were reactive in the ELISA showed 4-fold or greater changes in titer in H1- and H3-specific hemagglutination inhibition and microneutralization assays, which indicates they had not recently been infected with human influenza. None of these 4 healthcare workers reported any illness or potential exposure to H5N1 other than to patient A or B. The ELISA results were considered nonspecific. Paired serum samples from patient A showed clear seroconversion in both ELISA and H5 microneutralization. Serum specimens were not available from patient B.

We found no transmission of H5N1 to healthcare workers, despite the lack of infection control measures, which suggests inefficient human-to-human H5N1 transmission; similar results were found in Hanoi (5). Droplet and contact transmission are considered the most effective means of transmitting influenza A in hospitals, and the clinical importance of airborne transmission has not been fully elucidated (6). Diarrhea in H5N1-infected patients potentially contains viable virus (1,7) and may affect the H5N1 transmission route. While these results appear reassuring, the limited options that were available to prevent nosocomial infection are worrisome. If reassortment between avian and human influenza A virus were to occur, resulting in a virus with pandemic potential, nosocomial transmission would be a concern. Infection control measures are crucial in all cases of avian influenza, and resources to prevent nosocomial infection must be made available in affected countries.

**Table:** 



